# Agreement of different OCT scan directions for individual retinal-layer thickness measurements in multiple sclerosis subjects with prior unilateral optic neuritis

**DOI:** 10.1038/s41598-021-04611-y

**Published:** 2022-01-12

**Authors:** Alberto Domínguez-Vicent, Maria Nilsson, Rune Brautaset, Abinaya Priya Venkataraman

**Affiliations:** Division of Eye and Vision, Department of Clinical Neuroscience, Karolinska Institute, 171 77 Stockholm, Sweden

**Keywords:** Diseases, Health care, Medical research

## Abstract

The similarities between horizontal and vertical Optical Coherence Tomography (OCT) scans for the individual retinal layer thickness measurements in the macula was evaluated. Two volumetric scans (B-scans oriented horizontally and vertically) were performed in 64 multiple sclerosis subjects with history of unilateral optic neuritis and 64 healthy controls. The agreement between the thickness measurements with horizontal and vertical OCT scans was evaluated in 3 groups of eyes: healthy controls, eyes with history of optic neuritis and the fellow eyes. The mean difference in individual layer thickness between the scans was smaller than the instrument’s axial resolution in all 3 groups. The limit of agreement (LoA) varied among the different layers and sectors analyzed and this trend was similar in all the groups. For the inner retinal layers (retinal nerve fiber layer to inner nuclear layer), the inner macular sectors had a larger LoA compared to the corresponding outer sectors. In the outer plexiform and nuclear layers, the central and inner sectors (except inner temporal) had LoA larger than the other sectors and layers. The larger LoA seen for different layers and sectors suggests that the scan direction must be same for the follow-up OCT measurements and in clinical studies.

## Introduction

Macular volumetric measurements using Optical Coherence Tomography (OCT) plays an important role in the diagnosis and follow up of various ocular and neurological disorders^1–4^. The OCTs that are clinically available are incorporated with their own segmentation algorithm which provides data of the total retinal thickness and individual layer thickness of ganglion cell layer (GCL), inner plexiform layer (IPL) and retinal nerve fiber layer (RNFL). Recent developments in instrumentation and image analysis algorithms allow further segmentation of individual layers, which is of great use in different areas in ophthalmology and neurology^5–7^.

The automated segmentation algorithm’s reliability for thickness measurement of RNFL, GCL and IPL have been widely studied in the past and are shown to be clinically acceptable^8–11^. Changes in these layers are shown to be a hallmark in the diagnosis of glaucoma^12–15^. The changes in the thickness of the inner retinal layers are reported in different neurologic disorders including multiple sclerosis^16,17^ and Parkinson’s disease^18,19^. In multiple sclerosis subjects, the retinal morphological changes are limited to the inner retinal layers in the absence of optic neuritis or when there is a good visual acuity recovery following optic neuritis; in the presence of optic neuritis with poor visual recovery, the changes can be seen in both inner and outer retinal layers can be seen. Another study has also shown that there are changes in the outer nuclear layer (ONL) that reverts to the baseline in eyes with optic neuritis^20^.

The reliability of the thickness measurements depends on various factors like scan resolution, acquisition time, segmentation algorithm and the retinal condition^8,9,21–23^. The scan direction is also shown to affect the precision of the quantitative measurements in both macula and optic disc^7,10,24^. For the total retinal measurements in the macular region, the horizontally oriented B-scans showed better repeatability for nasal and temporal sectors and the vertical scans showed better repeatability for superior and inferior sectors in healthy eyes^10^. For the peripapillary RNFL measurements, similar trend was reported in healthy eyes^24^. The similarities in the retinal layer thickness measurement between different scan directions were also evaluated in healthy subjects and similar results have been reported in a small cohort of multiple sclerosis subjects.

The variations in the individual retinal layer thickness measurement with different scan directions in multiple sclerosis subjects with history of optic neuritis could be different from healthy controls as the retinal morphology is different between the groups. In order to evaluate this, we included multiple sclerosis subjects with unilateral optic neuritis in the present study, so that the variations in the fellow eye can also be studied. The agreement between the measurement of individual retinal layer thickness with the two scan directions are evaluated in 3 sub-groups: healthy controls, eyes with a history of optic neuritis and the fellow eyes.

## Methods

### Subjects

OCT measurement data from 64 multiple sclerosis subjects with history of unilateral optic neuritis was retrieved for this study. All subjects were measured at the Neurology Clinic, Neuro Centrum at Karolinska University Hospital, Solna, Sweden. Subjects with acute optic neuritis within 6 months before the OCT measurements were excluded. Subjects with any other neurologic or ocular diseases that could influence the outcome measures were excluded. 64 age matched healthy controls were also included in this study. The study design followed the Declaration of Helsinki principles and was approved by the the Regional Ethical Committee (Regionala etikpröningsnämden, Stockholm 2011/874-31/2). Informed consents were obtained from all participants.

### OCT measurements

**Figure 1:**
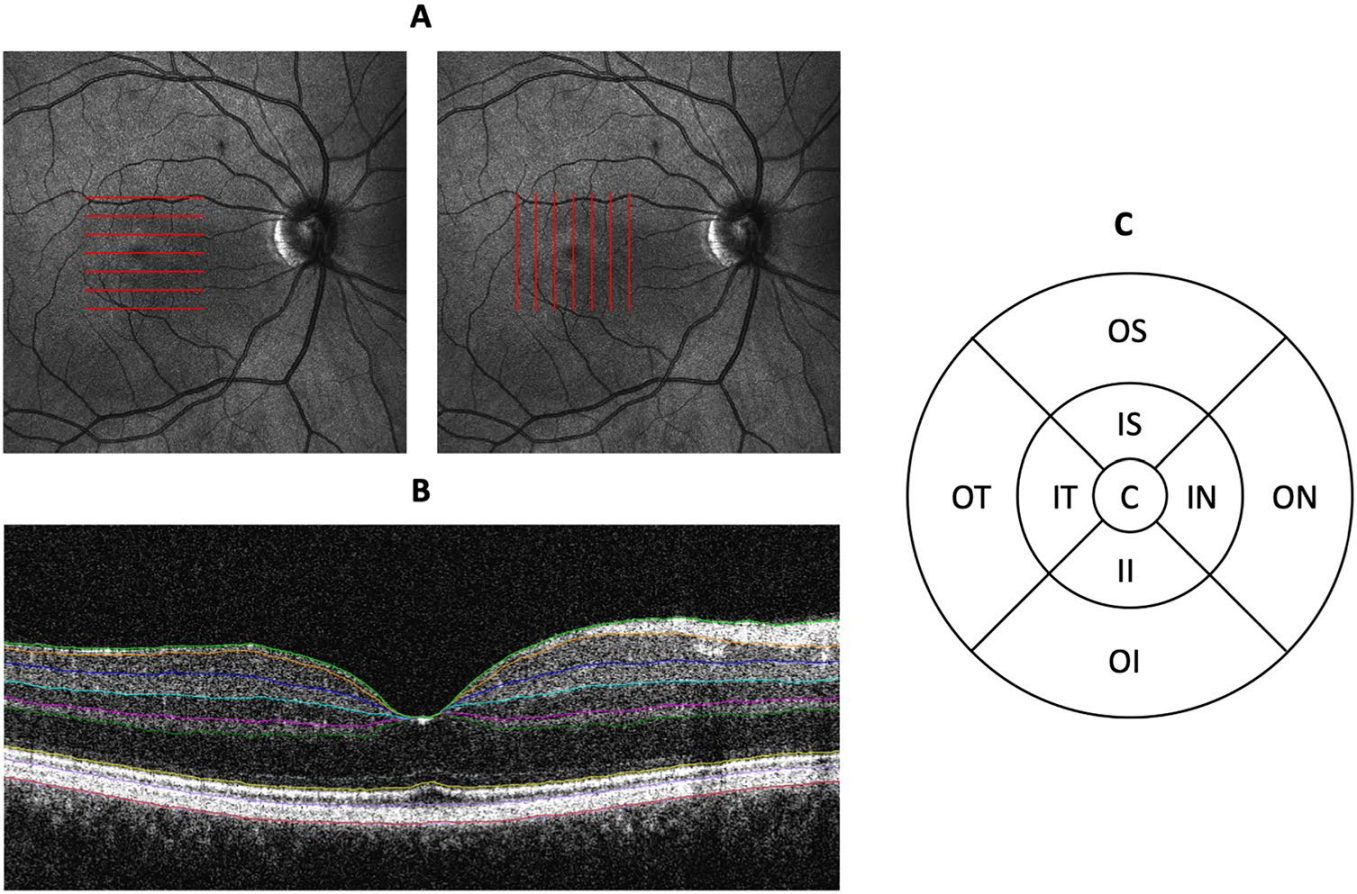
Macular volumetric OCT measurements. (**A**) Schematic representation of the B-scan orientation used in the macular volumetric measurements in the right eye. (**B**) An example B-scan showing the individual retinal layer segmentation. (**C**) Schematic representation of the ETDRS sectors. *C* central circle, *II* inner inferior, *OI* outer inferior, *IN* inner nasal, *ON* outer nasal, *IS* inner superior, *OS* outer superior, *IT* inner temporal, *OT* outer temporal sectors.

All subjects underwent OCT examination using the Canon OCT HS-100 (Canon Europe, Netherlands) which performs up to 70,000 A-scans/second with an axial resolution of 3 µm. For the multiple sclerosis subjects, both eyes were included: eye with history of optic neuritis (ON), and the fellow eye with no history of optic neuritis (NON). For the healthy controls (HC), only one eye was included (33 right eyes and 31 left eyes). Two macular volumetric scans were performed with a scan area of 10 × 10 mm containing 128 B-scans each consisting of 1,024 A-scans. One volumetric scan had the B-scans oriented in horizontal direction and the other had the B-scans oriented in vertical direction (Fig. 1A). Only scans with signal strength of 7 or higher were included.

The automated segmentation software (version 4.5) of the instrument was used to delineate 8 different layers of the retina (Fig. 1B): RNFL, GCL, IPL, Inner Nuclear Layer (INL), Outer Plexiform Layer (OPL), ONL, Outer Segment (OS) and Retinal Pigment Epithelium (RPE). No manual adjustment of the segmentation was performed. The thickness of each layer specified above was evaluated in 9 sectors centered in the fovea based on Early Treatment Diabetic Retinopathy Study (ETDRS) protocol with the central circle, inner ring (Superior, Nasal, Inferior and temporal), and outer ring (Superior, Nasal, Inferior and temporal) with diameter of 1, 3, and 6 mm respectively (Fig. 1C).

### Statistical analysis

The agreement between macular volumetric measurements obtained from the horizontal and vertical B-scans were evaluated with Bland–Altman analyses for single measurement^25^. The agreement analysis was performed for each of the eight individual retinal layers in each of the nine sectors for the 3 groups (HC, ON and NON).

## Results

The study population includes 64 healthy controls (47 females) and 64 multiple sclerosis (46 females). The average age of the multiple sclerosis subjects and healthy controls were 44 ± 10 and 44 ± 15 years respectively. The average disease duration in multiple sclerosis subjects was 14 ± 8 years.

The average individual layer thickness in the 9 ETDRS sector for the three groups analyzed are shown in Table 1. The results from the Bland–Altman analyses macular volumetric measurements obtained from the horizontal and vertical B-scans for all the layers in nine ETDRS sectors are shown in Table 2. For the control eyes, the mean difference was less than 1 μm for all the layers in all the sectors. For the ON eyes, the mean difference was less than 1.5 μm for all the layers in all the sectors except the ONL in the central sector where the difference was 2.89 μm. For the fellow NON eyes, the mean difference was less than 1.5 μm for all the layers in all the sectors.

**Table 1:** Average thickness values for eight layers in nine early treatment diabetic retinopathy study sectors for the three groups.

Retinal layer	Eyes	Central		Inner inferior		Outer inferior		Inner nasal		Outer nasal		Inner superior		Outer superior		Inner temporal		Outer temporal	
		H	V	H	V	H	V	H	V	H	V	H	V	H	V	H	V	H	V
RNFL	HC	8.9 ± 2.0	8.8 ± 1.8	26.1 ± 3.8	26.7 ± 3.3	40.0 ± 6.8	40.2 ± 6.6	21.2 ± 2.5	20.5 ± 2.2	48.3 ± 7.4	48.6 ± 7.5	24.7 ± 3.1	24.7 ± 3.1	38.4 ± 5.9	37.9 ± 6.0	17.2 ± 1.3	16.8 ± 1.6	18.3 ± 1.4	18.3 ± 1.6
	ON	9.7 ± 3.5	9.6 ± 2.6	20.3 ± 3.4	21.3 ± 3.9	29.9 ± 7.6	30.3 ± 7.8	19.0 ± 3.6	18.3 ± 2.4	36.3 ± 9.0	36.0 ± 8.7	20.4 ± 3.3	20.7 ± 3.3	29.1 ± 6.5	29.2 ± 6.5	17.1 ± 2.9	16.5 ± 2.1	17.5 ± 1.9	17.4 ± 1.8
	NON	8.9 ± 2.0	9.4 ± 1.9	23.5 ± 3.8	24.4 ± 3.7	35.5 ± 7.3	35.8 ± 7.1	20.7 ± 3.5	20.1 ± 2.9	42.6 ± 8.6	42.5 ± 9.4	22.6 ± 3.7	23.1 ± 3.6	34.0 ± 6.4	33.8 ± 6.2	17.4 ± 1.6	17.0 ± 1.8	18.3 ± 1.9	18.2 ± 2.4
GCL	HC	16.7 ± 5.1	16.6 ± 4.6	50.9 ± 5.3	50.5 ± 5.4	28.0 ± 3.1	27.7 ± 2.8	51.6 ± 5.7	51.8 ± 5.8	34.2 ± 4.3	34.2 ± 4.1	52.0 ± 5.8	51.4 ± 5.3	29.9 ± 3.2	30.4 ± 3.0	48.9 ± 5.0	48.7 ± 4.9	32.2 ± 3.6	31.9 ± 3.9
	ON	13.0 ± 4.9	13.0 ± 4.9	36.2 ± 9.6	35.4 ± 8.8	24.8 ± 2.9	24.8 ± 2.7	36.6 ± 9.8	36.4 ± 9.5	27.4 ± 4.5	27.1 ± 5.2	36.5 ± 8.9	36.2 ± 8.8	25.5 ± 3.5	25.9 ± 3.3	33.2 ± 9.3	33.4 ± 9.4	26.3 ± 4.4	26.2 ± 4.3
	NON	15.3 ± 4.9	15.6 ± 5.2	43.2 ± 9.0	42.7 ± 8.6	26.4 ± 3.0	26.3 ± 3.1	43.4 ± 9.7	43.0 ± 9.6	29.9 ± 4.5	29.8 ± 4.9	43.4 ± 9.0	43.1 ± 8.8	27.3 ± 3.3	27.7 ± 3.4	41.2 ± 8.9	41.2 ± 9.1	29.0 ± 4.2	28.9 ± 4.5
IPL	HC	16.4 ± 4.2	16.4 ± 3.9	41.6 ± 3.8	41.6 ± 3.3	32.1 ± 2.9	31.9 ± 3.0	40.7 ± 3.4	40.6 ± 3.3	35.6 ± 3.2	34.8 ± 3.2	41.1 ± 3.2	40.4 ± 3.3	33.0 ± 3.0	33.2 ± 3.1	39.0 ± 3.6	39.3 ± 3.8	36.5 ± 3.3	35.6 ± 3.2
	ON	15.5 ± 4.8	15.0 ± 4.1	36.3 ± 4.8	36.4 ± 4.8	29.5 ± 3.1	29.1 ± 3.2	36.2 ± 5.0	36.2 ± 4.7	31.2 ± 4.2	31.1 ± 3.9	36.9 ± 4.8	36.4 ± 4.3	30.7 ± 3.2	30.5 ± 3.2	33.9 ± 5.2	34.1 ± 5.0	31.8 ± 4.2	32.0 ± 3.9
	NON	16.1 ± 4.1	16.3 ± 4.0	38.6 ± 4.8	38.6 ± 4.8	31.1 ± 3.4	30.7 ± 3.6	37.9 ± 4.6	37.8 ± 5.2	33.3 ± 4.6	33.0 ± 4.5	38.8 ± 4.6	37.9 ± 4.5	31.9 ± 3.3	32.1 ± 3.3	36.4 ± 4.8	36.5 ± 5.1	34.5 ± 4.1	34.2 ± 4.2
INL	HC	17.7 ± 4.7	17.7 ± 4.3	43.1 ± 2.9	43.0 ± 2.9	32.1 ± 2.7	31.9 ± 2.4	43.0 ± 3.3	43.5 ± 3.2	35.7 ± 2.5	35.9 ± 2.4	43.2 ± 2.9	43.4 ± 2.5	33.2 ± 2.5	33.8 ± 2.2	40.1 ± 2.7	40.5 ± 2.9	35.1 ± 2.6	35.2 ± 2.6
	ON	18.3 ± 5.9	17.5 ± 4.8	42.5 ± 3.3	42.4 ± 3.6	31.8 ± 3.1	31.4 ± 3.1	42.6 ± 4.3	43.2 ± 3.7	34.6 ± 3.8	34.4 ± 3.8	43.0 ± 3.3	42.6 ± 3.4	32.8 ± 3.0	33.2 ± 2.9	39.3 ± 3.3	39.5 ± 3.5	34.7 ± 3.1	34.7 ± 3.2
	NON	18.2 ± 4.9	18.4 ± 4.4	43.1 ± 3.5	43.4 ± 3.8	32.4 ± 3.0	32.2 ± 3.1	43.2 ± 4.4	43.6 ± 4.4	35.2 ± 3.8	35.1 ± 3.9	43.4 ± 3.5	43.4 ± 3.5	33.5 ± 3.2	33.8 ± 3.1	40.0 ± 3.4	40.5 ± 3.5	35.4 ± 3.2	35.4 ± 3.4
OPL	HC	18.7 ± 6.1	18.5 ± 5.9	32.9 ± 7.0	32.7 ± 5.5	25.4 ± 3.0	24.9 ± 2.4	34.2 ± 8.7	34.4 ± 9.0	28.5 ± 4.5	28.8 ± 4.6	32.9 ± 5.9	33.1 ± 5.3	25.9 ± 2.9	25.7 ± 2.7	28.9 ± 3.4	28.9 ± 3.3	25.0 ± 2.2	25.2 ± 2.2
	ON	19.2 ± 6.4	18.0 ± 5.8	31.2 ± 3.9	31.1 ± 3.5	25.3 ± 3.0	25.1 ± 2.8	35.2 ± 9.3	34.6 ± 8.6	28.9 ± 4.6	29.2 ± 4.8	31.3 ± 4.7	31.0 ± 4.4	25.4 ± 2.6	25.3 ± 2.4	27.1 ± 2.2	26.8 ± 2.2	24.5 ± 1.8	24.8 ± 2.2
	NON	18.5 ± 6.1	19.0 ± 5.6	32.4 ± 4.8	32.7 ± 5.9	26.0 ± 3.2	25.6 ± 3.3	34.8 ± 8.0	36.3 ± 9.1	29.2 ± 4.5	29.8 ± 4.7	31.3 ± 5.1	31.7 ± 5.7	25.3 ± 2.3	25.4 ± 2.6	27.3 ± 3.1	27.3 ± 2.9	24.6 ± 1.6	24.8 ± 1.6
ONL	HC	123.3 ± 9.4	123.5 ± 9.8	85.4 ± 10.8	85.3 ± 10.3	69.7 ± 6.7	69.9 ± 6.3	91.9 ± 12.3	91.7 ± 12.3	74.4 ± 7.1	74.3 ± 7.2	88.2 ± 11.6	88.8 ± 11.8	78.6 ± 6.8	79.0 ± 7.2	94.5 ± 9.2	94.1 ± 8.8	76.9 ± 6.9	76.6 ± 7.0
	ON	120.4 ± 12.2	123.3 ± 9.5	89.4 ± 10.1	88.8 ± 9.8	71.8 ± 6.6	71.6 ± 6.6	91 ± 14.7	91.5 ± 13.3	74.6 ± 8.5	74.7 ± 8.4	91.8 ± 10.8	92.6 ± 9.9	80.3 ± 7.0	80.7 ± 7.0	97.9 ± 8.2	98.0 ± 8.0	78.5 ± 6.7	78.5 ± 6.5
	NON	122.4 ± 9.3	122.8 ± 10.1	87.3 ± 9.7	86.1 ± 10.7	70.9 ± 6.7	70.6 ± 6.8	90.4 ± 12.3	89.2 ± 13.1	74.2 ± 7.6	74.1 ± 7.8	91.3 ± 10.0	91.3 ± 11.1	80.4 ± 7.0	80.6 ± 7.3	97.1 ± 8.4	96.6 ± 8.5	78.4 ± 6.2	78.5 ± 6.2
OS	HC	33.8 ± 1.7	33.7 ± 1.6	28.5 ± 1.7	28.3 ± 1.7	30.5 ± 2.6	30.2 ± 2.7	29.7 ± 1.5	29.8 ± 1.7	28.8 ± 2.3	28.8 ± 2.4	29.0 ± 1.7	29.0 ± 1.6	29.2 ± 2.7	29.0 ± 2.6	29.2 ± 1.4	29.0 ± 1.5	29.0 ± 2.6	29.0 ± 2.6
	NON	34.7 ± 1.5	34.6 ± 1.5	29.1 ± 2.1	29.3 ± 2.0	30.4 ± 2.2	30.6 ± 2.2	30.2 ± 1.6	30.3 ± 1.8	28.8 ± 2.1	28.9 ± 2.0	29.5 ± 1.8	29.7 ± 1.8	29.0 ± 2.3	29.3 ± 2.1	29.6 ± 1.7	29.7 ± 1.6	29.3 ± 2.7	29.5 ± 2.6
	ONL	34.7 ± 1.5	34.6 ± 1.6	29.3 ± 2.2	29.2 ± 2.0	30.4 ± 2.4	30.7 ± 2.1	30.1 ± 1.8	30.3 ± 1.8	28.9 ± 2.1	28.7 ± 2.1	29.7 ± 2.0	29.8 ± 1.8	29.1 ± 2.4	28.9 ± 2.2	29.9 ± 1.9	29.7 ± 2.2	29.7 ± 3.0	29.6 ± 2.9
RPE	HC	35.8 ± 1.9	35.8 ± 1.6	33.0 ± 1.9	33.0 ± 2.0	28.3 ± 2.8	28.5 ± 2.8	33.7 ± 1.8	33.5 ± 1.9	30.9 ± 2.5	30.4 ± 2.9	33.5 ± 2.0	33.6 ± 1.9	31.3 ± 2.8	31.3 ± 2.8	33.5 ± 1.9	33.4 ± 1.9	31.3 ± 3.0	30.9 ± 3.0
	ON	37.0 ± 1.3	36.8 ± 1.4	34.0 ± 2.4	33.6 ± 2.5	29.5 ± 2.5	29.2 ± 2.1	34.3 ± 2.0	34.0 ± 2.0	31.9 ± 2.8	31.8 ± 2.9	34.3 ± 2.3	34.2 ± 2.3	32.6 ± 2.7	32.7 ± 2.5	34.5 ± 2.1	34.0 ± 2.2	32.1 ± 2.9	31.2 ± 3.0
	NON	36.9 ± 1.5	36.8 ± 1.4	33.7 ± 2.5	33.2 ± 2.6	29.1 ± 2.4	28.8 ± 2.3	33.9 ± 1.8	34.0 ± 2.2	31.4 ± 2.4	31.2 ± 2.6	34.3 ± 2.3	34.2 ± 2.3	32.3 ± 2.6	32.1 ± 2.4	34.5 ± 2.3	33.8 ± 2.1	32.1 ± 2.9	31.1 ± 2.9

All values are expressed in microns.

*HC* healthy controls, *ON* eyes with optic neuritis, *NON* fellow eyes without optic neuritis, *H* horizontal scan, *V* vertical scan, *RNFL* retinal nerve fiber layer, *GCL* ganglion cell layer, *IPL* inner plexiform layer, *INL* inner nuclear layer, *OPL* outer plexiform layer, *ONL* outer nuclear layer, *OS* outer segment, *RPE* retinal pigment epithelium.

**Table 2:** Bland–Altmann analysis results.

Retinal layer	Eyes	Central	Inner inferior	Outer inferior	Inner nasal	Outer nasal	Inner superior	Outer superior	Inner temporal	Outer temporal
RNFL	HC	-0.05 [-3.55 to 3.453]	0.58 [− 3.08 to 4.24]	0.27 [− 2.62 to 3.131]	− 0.64 [− 3.4 to 2.12]	0.30 [− 4.03 to 4.63]	− 0.06 [− 3.89 to 3.76]	− 0.44 [− 3.18 to 2.31]	− 0.36 [− 2.58 to 1.86]	0.06 [− 1.45 to 1.58]
	ON	− 0.14 [− 6.52 to 6.24]	1.03 [− 2.87 to 4.94]	0.40 [− 2.75 to 3.53]	− 0.72 [− 7.30 to 5.87]	− 0.25 [− 6.60 to 6.10]	0.33 [− 2.95 to 3.61]	0.08 [− 2.95 to 3.11]	− 0.59 [− 5.48 to 4.29]	− 0.14 [− 2.75 to 2.47]
	NON	0.45 [− 3.57 to 4.48]	0.81 [− 4.15 to 5.77]	0.28 [− 3.34 to 3.90]	− 0.61 [− 6.19 to 4.97]	− 0.11 [− 6.95 to 6.73]	0.52 [− 3.34 to 4.37]	− 0.25 [− 3.51 to 3.01]	− 0.33 [− 3.29 to 2.64]	− 0.05 [− 3.24 to 3.14]
GCL	HC	− 0.13 [− 6.01 to 5.75]	− 0.41 [− 4.72 to 3.91]	− 0.30 [− 3.11 to 2.51]	0.19 [− 3.34 to 3.71]	0.03 [− 2.7 to 2.8]	− 0.63 [− 4.63 to 3.38]	0.53 [− 2.1 to 3.17]	− 0.19 [− 4.26 to 3.88]	− 0.23 [− 2.62 to 2.15]
	ON	− 0.03 [− 5.16 to 5.10]	− 0.81 [− 5.40 to 3.78]	0.03 [− 1.91 to 1.98]	− 0.23 [− 4.58 to 4.11]	− 0.23 [− 3.27 to 2.81]	− 0.25 [− 3.62 to 3.12]	0.41 [− 1.49 to 2.31]	0.23 [− 3.74 to 4.21]	− 0.09 [− 2.63 to 2.44]
	NON	0.31 [− 4.01 to 4.63]	− 0.48 [− 3.94 to 2.97]	− 0.14 [− 2.14 to 1.86]	− 0.41 [− 4.92 to 4.10]	− 0.09 [− 3.36 to 3.18]	− 0.31 [− 3.49 to 2.87]	0.36 [− 1.69 to 2.41]	0.03 [− 3.81 to 3.87]	− 0.06 [− 3.55 to 3.43]
IPL	HC	− 0.03 [− 4.65 to 4.59]	− 0.06 [− 4.41 to 4.28]	− 0.25 [− 2.54 to 2.04]	− 0.11 [− 3.72 to 3.51]	− 0.81 [− 3.23 to 1.60]	− 0.64 [− 4.31 to 3.02]	0.266 [− 2 to 2.54]	0.328 [− 3.34 to 4.00]	− 0.86 [− 2.83 to 1.11]
	ON	− 0.45 [− 5.47 to 4.57]	0.09 [− 3.01 to 3.19]	− 0.38 [− 2.49 o 1.74]	0.00 [− 3.78 to − 3.78]	− 0.09 [− 2.79 to 2.61]	− 0.48 [− 3.57 to 2.60]	− 0.16 [− 2.13 to 1.83]	0.20 [− 3.72 to 4.13]	0.19 [− 2.30 to 2.68]
	NON	0.19 [− 3.70 to 4.07]	− 0.02 [− 3.96 to 3.93]	− 0.44 [− 2.72 to 1.85]	− 0.11 [− 3.89 to 3.67]	− 0.23 [− 3.15 to 2.68]	− 0.95 [− 4.64 to 2.73]	0.17 [− 1.63 to 1.97]	0.08 [− 3.30 to 3.45]	− 0.34 [− 3.43 to 2.74]
INL	HC	0.02 [− 4.82 to 4.85]	− 0.03 [− 3.13 to 3.07]	− 0.22 [− 2.62 to 2.187]	0.50 [− 2.09 to 3.09]	0.23 [− 1.39 to 1.86]	0.27 [− 2.49 to 3.02]	0.66 [− 1.36 to 2.80]	0.42 [− 2.39 to 3.23]	0.09 [− 1.61 to 1.79]
	ON	− 0.81 [− 6.63 to 5.01]	− 0.19 [− 3.04 to 2.67]	− 0.39 [− 2.16 to 1.38]	0.56 [− 4.08 to 5.21]	− 0.17 [− 2.84 to 2.50]	− 0.38 [− 2.34 to 1.59]	0.34 [− 1.58 to 2.26]	0.19 [− 2.85 to 3.23]	0.02 [− 2.96 to 2.99]
	NON	0.16 [− 4.56 to 4.87]	0.28 [− 3.29 to 3.85]	− 0.23 [− 2.14 to 1.67]	0.39 [− 3.84 to 4.62]	− 0.09 [2.34 to − 2.53]	− 0.05 [− 2.91 to 2.82]	0.27 [1.84 to − 1.31]	0.48 [− 2.48 to 3.45]	− 0.06 [− 2.06 to 1.94]
OPL	HC	− 0.20 [− 6.15 to 5.75]	− 0.20 [− 10.2 to 9.81]	− 0.50 [− 4.24 to 3.25]	0.19 [− 7.73 to 7.10]	0.28 [− 2.97 to 3.53]	0.188 [− 9.93 to 10.3]	− 0.13 [− 4.58 to 4.32]	0.02 [− 4.54 to 4.576]	0.25 [− 2.07 to 2.56]
	ON	− 1.20 [− 7.21 to 4.81]	− 0.08 [− 4.43 to 4.27]	− 0.28 [− 2.91 to 2.34]	− 0.56 [− 9.91 to 8.78]	0.34 [− 2.92 to 3.61]	− 0.33 [− 7.52 to 6.86]	− 0.13 [− 3.08 to 2.83]	− 0.25 [− 2.82 to 2.32]	0.29 [− 2.72 to 3.32]
	NON	0.48 [− 9.59 to 10.56]	0.34 [− 7.62 to 8.31]	− 0.34 [− 3.12 to 2.43]	1.44 [− 8.60 to 11.48]	0.61 [− 2.98 to 4.20]	0.41 [− 9.79 to 10.60]	0.03 [− 3.46 to 3.52]	0.02 [− 3.66 to 3.69]	0.20 [− 2.06 to 2.47]
ONL	HC	0.20 [− 12.10 to 12.55]	− 0.08 [− 11.7 to 11.5]	0.20 [− 4.18 to 4.58]	− 0.14 [− 8.32 to 8.04]	− 0.09 [− 4.16 to 3.98]	0.67 [− 11.6 to 12.92]	0.39 [− 4.65 to 5.44]	− 0.44 [− 5.91 to 5.04]	− 0.3 [− 2.73 to 2.14]
	ON	2.89 [− 13.43 to 19.21]	− 0.55 [− 6.79 to 5.70]	− 0.19 [− 2.98 to 2.60]	0.47 [− 10.12 to 11.06]	0.13 [− 3.48 to 3.73]	0.88 [− 7.17 to 8.92]	0.45 [− 2.43 to 3.33]	0.06 [− 4.27 to 4.39]	0.02 [− 3.38 to 3.41]
	NON	0.38 [− 16.46 to 17.21]	− 1.16 [− 11.42 to 9.10]	− 0.30 [− 3.71 to 3.12]	− 1.20 [− 13.92 to 11.51]	− 0.14 [− 3.82 to 3.54]lePara>	0.08 [− 13.01 to 13.17]	0.23 [− 3.86 to 4.33]	− 0.47 [− 5.05 to 4.11]	0.13 [− 1.93 to 2.18]
OS	HC	− 0.11 [− 1.55 to 1.34]	− 0.19 [− 2.36 to 1.99]	− 0.30 [− 2.50 to 1.90]	0.04 [− 1.88 to 1.97]	0.06 [− 3.15 to 3.28]	0.03 [− 2.00 to 2.67]	− 0.14 [− 2.45 to 2.17]	− 0.25 [− 1.85 to 1.35]	− 0.06 [− 3.10 to 2.97]
	NON	− 0.11 [− 1.93 to 1.71]	− 0.03 [− 1.85 to 1.78]	0.28 [− 1.99 to 2.56]	0.11 [− 1.90 to 2.12]	− 0.14 [− 1.92 to 1.64]	0.09 [− 1.75 to 1.93]	− 0.19 [− 2.06 to 1.69]	− 0.17 [− 2.13 to 1.79]	− 0.17 [− 2.42 to 2.08]
	ONL	− 0.06 [− 2.30 to 2.17]	0.19 [− 2.87 to 3.25]	0.22 [− 1.83 to 2.27]	0.09 [− 2.84 to 3.03]	0.16 [− 2.19 to 2.50]	0.16 [− 2.17 to 2.48]	0.36 [− 2.57 to 3.29]	0.05 [− 2.12 to 2.21]	0.17 [− 3.15 to 3.50]
RPE	HC	0.02 [− 2.15 to 2.18]	− 0.02 [− 2.10 to 2.07]	0.25 [− 2.20 to 2.70]	− 0.17 [− 1.97 to 1.63]	− 0.45 [− 3.04 to 2.14]	0.05 [− 2.25 to 2.35]	− 0.03 [− 2.40 to 2.34]	− 0.09 [− 2.29 to 2.11]	− 0.42 [− 3.30 to 2.45]
	ON	− 0.27 [− 1.84 to 1.31]	− 0.38 [− 2.14 to 1.39]	− 0.31 [− 2.65 to 2.03]	− 0.20 [− 2.30 to 1.89]	− 0.17 [− 2.23 to 1.88]	− 0.13 [− 1.92 to 1.67]	0.06 [− 2.20 to 2.32]	− 0.56 [− 2.37 to 1.25]	− 0.95 [− 3.20 t 1.29]
	NON	− 0.16 [− 2.43 to 2.11]	− 0.48 [− 3.72 to 2.75]	− 0.27 [− 2.59 to 2.06]	0.13 [− 2.26 to 2.51]	− 0.20 [− 2.63 to 2.22]	− 0.03 [− 2.15 to 2.09]	− 0.25 [− 3.43 to 2.93]	− 0.63 [− 3.06 to 1.81]	− 1.03 [− 4.42 to 2.35]

Mean difference and limits of agreement for the horizontal and vertical scans.

All values are expressed in microns.

*HC* healthy controls, *ON* eyes with optic neuritis, *NON* fellow eyes without optic neuritis, *RNFL* retinal nerve fiber layer, *GCL* ganglion cell layer, *IPL* inner plexiform layer, *INL* inner nuclear layer, *OPL* outer plexiform layer, *ONL* outer nuclear layer, *OS* outer segment, *RPE* retinal pigment epithelium.

**Figure 2:**
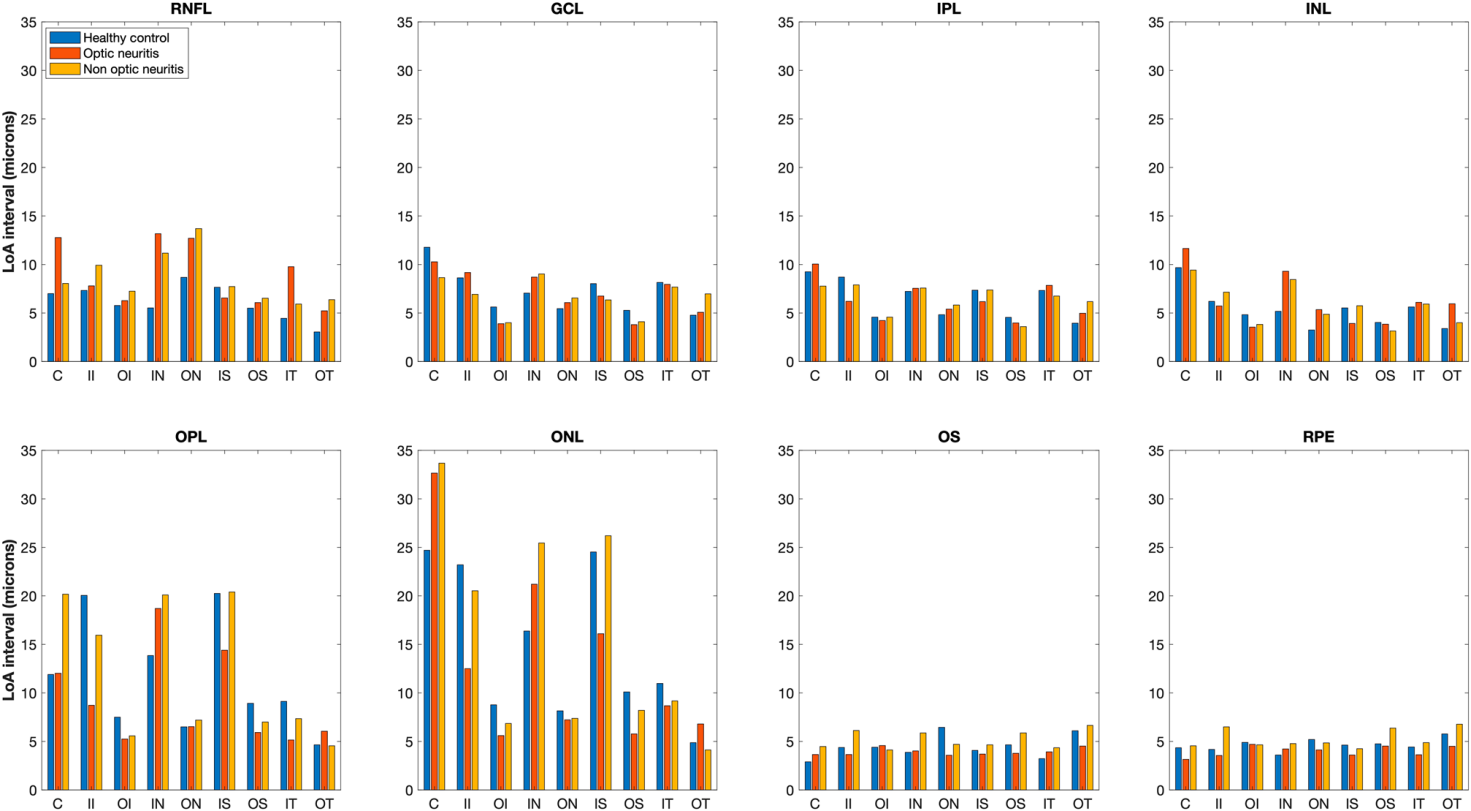
Limits of agreement (LoA) interval for eight layers in nine Early Treatment Diabetic Retinopathy Study sectors for the healthy controls (blue bars), eyes with optic neuritis (red bars), and fellow eyes without optic neuritis (orange bars). *C* central circle, *II* inner inferior, *OI* outer inferior, *IN* inner nasal, *ON* outer nasal, *IS* inner superior, *OS* outer superior, *IT* inner temporal, *OT* outer temporal sectors, *RNFL* retinal nerve fiber layer, *GCL* ganglion cell layer, *IPL* inner plexiform layer, *INL* inner nuclear layer, *OPL* outer plexiform layer, *ONL* outer nuclear layer, *OS* outer segment, *RPE* retinal pigment epithelium.

Figure 2 shows the limits of agreement (LoA) interval for all the layers in nine ETDRS sectors. There were large variations in the agreement among different layers and different sectors.

For the inner retinal layers (RNFL, GCL, IPL and INL), the LOA interval never exceeded 15 μm. For these layers, the inner sectors had larger LOA interval compared to the respective outer sectors and this tendency was seen in all the groups. The central sectors also had larger LoA interval compared to the outer sectors in all three groups. For the OPL and ONL layers, the variability in LoA interval among the sectors was larger compared to the other layers. The central and inner sectors (except inner temporal) for OPL and ONL had LoA intervals larger than 10 μm for all 3 groups. Both OS and RPE had uniform LoA intervals among the sectors in all three groups and the LoA intervals never exceeded 7 μm.

## Discussions

We evaluated the agreement between two macular volumetric scans for the measurements of individual retinal layer thickness in 3 groups: healthy controls, eyes with history of unilateral optic neuritis in multiple sclerosis subjects and in the fellow eyes. The only difference between the two volumetric scans is the orientation of the B-scan. In all the 3 groups, the mean difference between the horizontal and vertical volumetric scan was smaller than the instrument’s axial resolution (3 μm) for each of the individual retinal layer measured. There was a wide variation in the LOA interval for different layers and sectors and this variation was seen in all 3 groups.

The reduction of around 15 μm in average macular RNFL thickness and GCL-IPL thickness were reported in eyes with prior optic neuritis in multiple sclerosis subjects compared to healthy controls^26,27^. The present results show that the measurements of these layers with two different scan modes can differ up to 11 μm and 14 μm in the HC and multiple sclerosis subjects respectively, in some of the ETDRS sectors. This suggests that we cannot interchange the volumetric measurements from the horizontal and vertical scans. It has been reported that the reduction in GCL-IPL thickness in multiple sclerosis subjects with prior optic neuritis follows a horseshoe-like thickness reduction pattern corresponding to inner sectors (except inner temporal)^26^. These are the sectors that had larger differences between the horizontal and vertical scanning modes in the present study. This finding can also be interesting in the diagnosis and follow up of glaucoma which also causes macular RNFL, GCL and IPL reduction^14,15^. It can be suggested that the scan direction should be considered for both clinical follow up and for comparison of results from different research studies.

The LoA interval from the present results also showed that OPL and ONL in the central and inner sectors have the widest agreement interval in all 3 groups. A previous study evaluated the individual retinal layer thicknesses repeatability in nine ETDRS sectors with horizontal scans and showed worse repeatability for the inner sectors compared to the outer sectors in these layers^5^. It is known that if one method of measurement has poor repeatability, the agreement between the two methods will be poor. If both methods have poor repeatability, the agreement will be even worse^25^. The present results show larger LoA for the inner sectors, which is similar to the larger repeatability limits reported previously for the inner sectors^5^. Therefore, the presents results could be explained by the poor repeatability in these sectors.

The orientation of B-scan is shown to have an impact in the segmentation of individual retinal layers, in particular the delineation of OPL as the Henle’s fiber layer visibility is dependent on the orientation of the scan^28,29^. Due to this, a vertical oriented B-scan is suggested to have a potential advantage over horizontally oriented B-scan. A previous study comparing vertical and horizontal macular scans in a smaller sample of healthy and multiple sclerosis subjects showed larger LoA interval for OPL and ONL compared to the other four layers (RNFL, GCL-IPL, OS and RPE) analyzed^7^. We also found similar results in the present study in all 3 groups where a sector-wise analysis was performed. Though changes in these layers are not a diagnostic biomarker in multiple sclerosis, transient changes can occur in the ONL following the onset of optic neuritis, which is shown to be a predictor on visual acuity outcome^20^. The ONL thickness is also an important quantitative measurement in other disease conditions like central serous chorioretinopathy^30^, inherited retinal disorders^31,32^, or history of prematurity^33^, where the present results should be considered. It would be interesting to evaluate different scan orientations in other ocular disorders as well. The present study did not include compensation for the magnification effect due to axial length differences. However, we believe that this will not have an impact on the present results as the comparison of the 2 different scan directions were performed on the same eye.

In conclusion, the mean differences between the horizontal and vertical macular volumetric scan modes to measure individual retinal layer thickness were smaller than the axial resolution of the instrument. However, the wide LoA interval obtained for different layers and sectors in HC, ON and NON groups, emphasizes the importance of using the same scan direction during follow-up visits and in clinical studies.
